# Robotic Extended Thymectomy in Late‐Onset Myasthenia Gravis: A 21‐Year Retrospective Cohort Study of 172 Patients

**DOI:** 10.1111/ene.70388

**Published:** 2025-11-05

**Authors:** Luyu Huang, Feng Li, Zhongmin Li, Hongbin Zhang, Aron Elsner, Julia Strauchmann, Marco Nicolas Andreas, Tomasz Dziodzio, Aina Lask, Jens Neudecker, Daipeng Xie, Lintong Yao, Shaowei Wu, Haiyu Zhou, Andreas Meisel, Jens‐C. Rueckert

**Affiliations:** ^1^ Department of Surgery, Competence Center of Thoracic Surgery Charité Universitätsmedizin Berlin Berlin Germany; ^2^ Department of Thoracic Surgery Guangdong Provincial People's Hospital (Guangdong Academy of Medical Sciences), Southern Medical University Guangzhou China; ^3^ Department of Thoracic Surgery The First Affiliated Hospital of Zhengzhou University Zhengzhou China; ^4^ Department of Biochemistry Zhongshan School of Medicine, Sun Yat‐Sen University Guangzhou China; ^5^ Department of Neurology With Experimental Neurology, Integrated Myasthenia Gravis Center, Neuroscience Clinical Research Center Charité University Medicine Berlin Berlin Germany

**Keywords:** extended thymectomy, late‐onset myasthenia gravis, myasthenia gravis, neurological outcomes, robotic‐assisted surgery

## Abstract

**Background:**

The safety and feasibility of robotic‐assisted (RATS) thymectomy for myasthenia gravis (MG) with onset age ≥ 50 years remain unverified, particularly in very late‐onset MG (V‐LOMG).

**Methods:**

Patients were classified into late‐onset MG (LOMG, 50–64) and very late‐onset MG (V‐LOMG ≥ 65) based on age of onset. Composite neurological remission (CNR) included complete stable remission (CSR), pharmacologic remission (PR), and minimal manifestations‐0 (MM‐0), while favorable outcomes comprised CNR and MM1‐3.

**Results:**

Among 1041 patients, 172 with MG onset at ≥ 50 years who underwent RATS extended thymectomy were included in the final analysis. The LOMG group comprised 104 patients (45.2% male), while the V‐LOMG group included 68 patients (60.3% male). V‐LOMG patients had more preoperative MG crises, shorter onset‐to‐thymectomy intervals, heavier thymic specimens, and less hyperplasia. In ocular‐onset MG, generalization was more frequent in LOMG than in V‐LOMG. No significant differences were found in other baseline characteristics, perioperative parameters, postoperative complications, and adverse composite outcomes. At a 5.1‐year mean follow‐up, the V‐LOMG group had slightly higher CSR (7.4% vs. 6.7%), CNR (16.2% vs. 11.5%), and favorable outcome rates (52.9% vs. 45.2%) than the LOMG group, with no statistical significance. Both groups, especially V‐LOMG (16.0 mg vs. 2.1 mg, *p* < 0.001), showed a significant corticosteroid dose reduction at the last follow‐up, confirming the steroid‐sparing effect of thymectomy.

**Conclusions:**

RATS extended thymectomy appears to be a safe and feasible treatment for patients with MG of onset at age ≥ 50 years, including those with V‐LOMG, demonstrating a significant steroid‐sparing effect while maintaining favorable neurological outcomes.

## Graphical abstract

This 21‐year retrospective cohort study evaluated the safety and long‐term outcomes of robotic extended thymectomy in 172 patients with late‐onset myasthenia gravis (onset age ≥ 50 years). Both late‐ and very late‐onset subgroups showed comparable perioperative outcomes and achieved favorable neurological remission. A significant corticosteroid‐sparing effect was observed at long‐term follow‐up, particularly in the very late‐onset group.

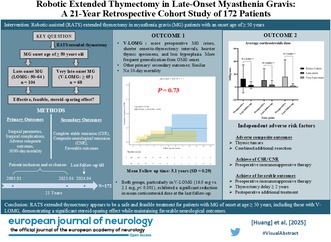

## 1 Introduction

Myasthenia gravis (MG) is a rare acquired autoimmune neuromuscular junction disorder primarily affecting voluntary muscles. Late‐onset MG (LOMG) is typically defined as onset after age 50 and is recognized as a distinct clinical subtype often associated with thymic atrophy [1]. LOMG often presents insidiously and is frequently overlooked or misdiagnosed due to comorbidities and age‐related physical decline [2]. Moreover, compared to early‐onset MG (EOMG), LOMG has a higher incidence, longer MG‐related absence duration, and greater MG‐related costs per patient year [3]. Therefore, this population warrants closer attention.

The first international randomized controlled trial (MGTX trial) [4, 5] confirmed the clinical efficacy of transsternal thymectomy in AChR‐Ab‐positive generalized MG patients. However, due to sample size limitations, the trial did not establish significant clinical benefits of thymectomy in MG patients over 50 years old.

Given the uncertain benefits of thymectomy in LOMG and the frailty and comorbidities common in elderly patients, current MG guidelines remain cautious [6, 7], particularly for very late‐onset MG (V‐LOMG, onset ≥ 65 years). Previous studies on LOMG surgery have been limited by small sample sizes, with most patients undergoing thymectomy via sternotomy. High‐quality, large‐scale evaluations of robotic‐assisted thoracic surgery (RATS) extended thymectomy in LOMG remain scarce, and its outcomes are not well defined.

This study aims to assess the short‐term postoperative and long‐term neurological outcomes of RATS extended thymectomy in MG patients with an onset age of ≥ 50 years, particularly in those with V‐LOMG.

## 2 Patients and Methods

This retrospective observational study included MG patients who underwent their first RATS extended thymectomy at this center, with an onset age of ≥ 50 years from January 2003 to April 2023, and follow‐up extending beyond April 2024. Patients were categorized into LOMG (50–64 years) and V‐LOMG (≥ 65 years) groups, and their outcomes were compared. The Institutional Review Board of Charité Universitätsmedizin Berlin approved the study (IRB: EA4/049/23) and waived the requirement for patient consent.

Exclusion criteria included: (I) nonrobotic or partial thymectomy; (II) postoperative follow‐up of less than 1 year; and (III) incomplete clinical data (e.g., missing Myasthenia Gravis Foundation of America (MGFA) classification, uncertain onset time, or unavailable drug dose comparisons).

An experienced multidisciplinary team (MDT) conducted a comprehensive preoperative evaluation [8] and symptom management [6] for MG patients, to ensure symptom stability and assess surgical suitability. RATS extended thymectomy entails the complete removal of the thymus, invaded adjacent structures, and all adipose tissue between the phrenic nerves, along with the thymic upper and lower poles. When thymomas adhere to the nerve, surgeons employ nerve‐sparing techniques to minimize neurological complications. The modified subxiphoid fourth trocar was evaluated and selectively used for large tumors. All specimens were photographed in situ, with the longest tumor diameter, specimen weight, and lymph nodes meticulously documented. Histological classification followed the World Health Organization (WHO) system [9].

Thoracic surgeons and neurologists conduct follow‐ups with patients every 3 to 6 months, adjusting medication type and dosage according to symptom progression. Final follow‐up data were collected from institutional medical records or obtained via structured telephone interviews.

The primary objective of this study was to evaluate the perioperative safety and feasibility of RATS extended thymectomy in MG patients with disease onset at age ≥ 50 years, particularly focusing on those with onset at age ≥ 65 years, who are historically considered higher risk. The corresponding primary outcome measures included perioperative parameters and short‐term postoperative outcomes (including adverse composite outcomes). Postoperative complications were categorized using the Clavien‐Dindo (C‐D) classification, with grade III or higher considered severe. Adverse composite outcomes incorporating intraoperative conversion, perioperative mortality, severe postoperative complications, positive margins, and postoperative MG deterioration.

The secondary outcome, focusing on neurological outcomes, was assessed using the Myasthenia Gravis Foundation of America Postintervention Status (MGFA‐PIS). To ensure a comprehensive evaluation, all patients had a follow‐up of over 1 year to determine the occurrence of complete stable remission (CSR), pharmacologic remission (PR), and minimal manifestations‐0 (MM‐0). Composite neurological remission (CNR) included CSR, PR, and MM‐0, and favorable outcomes included CNR and MM1‐3.

In addition to clinical status, we compared the number of medication types and the doses of immunosuppressive agents (prednisone and azathioprine [AZA]) and cholinesterase inhibitors between the preoperative period and the last follow‐up. These parameters were used as supplementary indicators to reflect neurological stability and the corticosteroid‐sparing effect after surgery.

### 2.1 Statistical Analysis

Categorical variables were compared using the Pearson chi‐square or Fisher's exact test, while continuous variables were analyzed using the Wilcoxon rank‐sum test. Paired sample *t*‐tests compared the mean medication dose and number of MG‐related medications before surgery and at the last follow‐up. Multivariate logistic regression (forward or bidirectional) was performed to identify independent risk factors for adverse composite outcomes, neurological outcomes, and favorable outcomes, adjusting for significant factors from the univariate analysis (*p* < 0.05). Kaplan–Meier (KM) analysis compared the cumulative incidence of CSR and CNR between groups, with differences assessed using the log‐rank test.

Statistical analyses were conducted using SPSS (version 27.0, IBM Corp, Armonk, NY, USA), GraphPad Prism (version 10.1.2, GraphPad Software, San Diego, CA), R (version 4.4, https://www.Rproject.org). The two‐sided *p*‐value < 0.05 was considered statistically significant.

No artificial intelligence (AI)–based tools were employed in this study, including data collection, analysis, interpretation, or manuscript preparation. All aspects of the research and writing were conducted exclusively by the authors.

## 3 Results

Among 1041 patients who underwent RATS extended thymectomy, 172 were included in the final analysis (Figure S1). The cohort comprised 104 patients in the LOMG group (60.5%, 45.2% male) and 68 in the V‐LOMG group (39.5%, 60.3% male) (Table 1).

**TABLE 1:** Clinical characteristics of LOMG patients stratified by age of onset in the cohorts.

Characteristics	Entire cohort	Late‐onset MG (≥ 50 to < 65)	Very‐late‐onset MG (≥ 65)	*p*
	*N* = 172	*n* = 104	*n* = 68	
Male sex	88 (51.2)	47 (45.2)	41 (60.3)	0.053
**Age of onset (year)**	**61.0 (55.0, 68.0)**	**56.0 (53.0, 60.0)**	**70.0 (67.0, 73.0)**	< 0.001**
Positive Antibody status	157 (91.3)	93 (89.4)	64 (94.1)	0.29
With OAID	36 (20.9)	22 (21.2)	14 (20.6)	0.93
With comorbidities	142 (82.6)	84 (80.8)	58 (85.3)	0.45
Preoperative MGFA classification				
I	40 (23.3)	25 (24.0)	15 (22.1)	0.36
II	107 (62.2)	63 (60.6)	44 (64.7)	
III	21 (12.2)	15 (14.4)	6 (8.8)	
IV	0 (0)	0 (0)	0 (0)	
V	4 (2.3)	1 (0.96)	3 (4.4)	
OMG at disease onset	94 (54.7)	60 (57.7)	34 (50.0)	0.32
**Generalization from OMG**	**71 (75.5)**	**48 (80.0)**	**23 (67.6)**	0.028*
OMG generalization before ThX	35 (49.3)	23 (47.9)	12 (52.2)	0.81
Preoperative therapy				
NT (No medication)	10 (5.8)	5 (4.8)	5 (7.4)	0.38
Prednisone/IM/prednisone +IM (immunosuppressive therapy only)	8 (4.7)	6 (5.8)	2 (2.9)	
Only cholinesterase inhibitors	42 (24.4)	30 (28.9)	12 (17.7)	
Cholinesterase inhibitors + prednisone/IM/prednisone +IM	97 (56.4)	54 (51.9)	43 (63.2)	
Treatment including PE/IG/OT	15 (8.7)	9 (8.7)	6 (8.8)	
Preoperative immunosuppressive therapy	119 (69.2)	68 (65.4)	51 (75.0)	0.18
**Preoperative MG crisis (impending or manifest)**	**22 (12.8)**	**9 (8.7)**	**13 (19.1)**	0.045*
**Time from symptom onset to ThX (months)**	**10.0** **(5.0, 21.3)**	**12.0** **(6.0, 26.3)**	**8.0** **(4.8, 16.5)**	0.011*
**Delay from onset to ThX** ≥ **1 year**	**79 (45.9)**	**55 (52.9)**	**24 (35.3)**	0.024*
**Delay from onset to ThX** ≥ **2 years**	**40 (23.3)**	**30 (28.9)**	**10 (14.7)**	0.032*
Delay from onset to ThX ≥ 5 years	6 (3.5)	6 (5.77)	0 (0)	0.11
Time from symptom onset to diagnosis (months)	3.0 (0.25, 9.0)	3.5 (1.0, 10.8)	2.0 (0.0, 5.0)	0.076
Delay from symptom onset to diagnosis ≥ 9 months	47 (27.3)	32 (30.8)	15 (22.1)	0.21

*Note:* Values are presented as median (Interquartile range, IQR) or number (%).

Abbreviations: GMG, Generalized myasthenia gravis; IG, IVIg therapy; IM, Immunosuppression therapy other than prednisone (azathioprine, mycophenolate mofetil, methotrexate); LOMG, late‐onset myasthenia gravis; MG, Myasthenia Gravis; MGFA, Myasthenia Gravis Foundation of America; NT, No therapy; OAID, Other auto‐immune diseases; OMG, Ocular myasthenia gravis; OT, Other forms of therapy (rituximab, eculizumab, efgartigimod, ravulizumab, daratumumab); PE, Plasma exchange therapy; ThX, Thymectomy.

*, Significant at *p* < 0.05; **, Significant at *p* < 0.005. Bolded rows denote clinical factors that showed statistically significant associations (*p* < 0.05) in the multivariable logistic regression analysis. This highlighting aims to distinguish independent predictors identified after adjustment for confounding variables.

The V‐LOMG patients had a higher incidence of preoperative impending or manifest MG crisis (*p* = 0.045). LOMG patients experienced a longer interval from symptom onset to thymectomy (12.0 vs. 8.0 months, *p* = 0.011) and were more likely to have a thymectomy delay of ≥ 1 year and ≥ 2 years (both *p* < 0.05) compared to V‐LOMG patients. In contrast, there was no significant difference between the two groups in the interval from symptom onset to diagnosis (3.5 vs. 2.0 months, *p* = 0.076). Using the 75th percentile of the entire cohort's diagnostic interval (≥ 9 months) to define delayed diagnosis, the proportion of patients with diagnostic delay did not significantly differ between groups.

In this cohort, 94 patients (54.7%) presented with ocular MG (OMG) at disease onset, of whom 71 (75.5%) experienced subsequent generalization. There was no significant difference between the LOMG and V‐LOMG groups in the proportion of OMG onset or prethymectomy generalization; however, the overall generalization rate from OMG was significantly higher in the LOMG group (*p* = 0.028). Figure S2B shows the distribution of preoperative MGFA clinical classifications between the two groups. Over 50% of patients in both groups initially presented with OMG symptoms, with preoperative MGFA classifications primarily in MGFA I (23.3%) and II (62.2%).

Figure S2A depicts the trend in RATS extended thymectomies performed on LOMG patients from 2003 to April 2023, along with the introduction timelines of three robotic system generations. Based on institutional practice, the year 2014 was selected as the cut‐off point to categorize the surgical era for further analysis.

Table 2 compares the surgical characteristics and perioperative outcomes between the two groups. V‐LOMG patients had heavier specimens (152 g vs. 119 g, *p* = 0.028) and a lower prevalence of thymic follicular hyperplasia (8.8% vs. 26.0%, *p* = 0.005). No significant differences were observed in the proportions of thymic atrophy, pathological subtypes of thymic tumors, or ectopic thymic tissue between the two groups. Among 52 thymic tumors in this study, only one was a thymic carcinoma, while the rest were thymomas. There were no significant differences between the two groups in postoperative therapy, postoperative additional postoperative plasma exchange (PE) + IVIg (IG) + other therapies (OT), or postoperative impending or manifest MG crisis (Table 2).

**TABLE 2:** Surgical characteristics and perioperative outcomes stratified by age of onset in the cohorts.

Characteristics	Entire cohort	Late‐onset MG (≥ 50 to < 65)	Very‐late‐onset MG (≥ 65)	*p*
	*N* = 172	*n* = 104	*n* = 68	
Operation year ≥ 2014	140 (81.4)	87 (83.7)	53 (77.9)	0.35
Surgical duration (min)	170.0 (140.0, 207.5)	170.0 (139.0, 203.0)	170.0 (142.5, 216.5)	0.51
Chest tube duration (days)	2.0 (2.0, 3.0)	2.0 (2.0, 3.0)	2.0 (2.0, 3.0)	0.46
Hospital LOS (days)	4.0 (4.0, 6.0)	4.0 (4.0, 6.0)	4.0 (4.0, 6.0)	0.85
Number of LND	5.0 (2.0, 10.0)	5.5 (2.3, 12.0)	5.0 (2.0, 7.0)	0.37
Conversion to sternotomy	2 (1.2)	0 (0)	2 (2.9)	0.16
Positive margin (R1)	2 (1.2)	1 (1.0)	1 (1.5)	> 0.99
Combined additional resection	9 (5.2)	6 (5.8)	3 (4.4)	0.97
**Weight of specimen (g)**	**128.0 (84.0, 204.3)**	**119.0 (74.5, 175.0)**	**152.0 (100.5, 214.5)**	0.028*
**Thymic hyperplasia**	**33 (19.2)**	**27 (26.0)**	**6 (8.8)**	0.005*
Thymic atrophy	37 (21.5)	20 (19.2)	17 (25.0)	0.37
Thymic tumor	52 (30.2)	31 (29.8)	21 (30.9)	0.88
Thymoma A	8 (15.4)	4 (12.9)	4 (19.0)	
Thymoma AB	12 (23.1)	7 (22.6)	5 (23.8)	
Thymoma B1	8 (15.4)	6 (19.4)	2 (9.5)	
Thymoma B2	14 (26.9)	8 (25.8)	6 (28.6)	
Thymoma B3	9 (17.3)	5 (16.1)	4 (19.0)	
Thymic carcinoma	1 (1.9)	1 (3.2)	0 (0)	
Low‐risk thymoma (types A, AB, B1)	28 (53.8)	17 (54.8)	11 (52.4)	0.96
Ectopic thymic tissue	24 (14.0)	14 (13.5)	10 (14.7)	0.83
Postoperative impending MG crisis or manifest crisis (≤ 1 month)	13 (7.6)	9 (8.7)	4 (5.9)	0.50
Postoperative impending MG crisis or manifest crisis (> 1 month)	20 (11.6)	14 (13.5)	6 (8.8)	0.35
Postoperative C‐D complication grade				
No complications (grade 0)	136 (79.1)	84 (80.8)	52 (76.5)	0.18
Minor complications (grade 1–2)	30 (17.4)	19 (18.3)	11 (16.2)	
Major complications (grade 3–5)	6 (3.5)	1 (0.96)	5 (7.4)	
Adverse composite outcomes^a^	12 (7.0)	5 (4.8)	7 (10.3)	0.28
Postoperative 30/90‐day mortality	0 (0)	0 (0)	0 (0)	> 0.99
Postoperative therapy				
NT (No medication)	15 (8.7)	8 (7.7)	7 (10.3)	0.43
Prednisone/IM/prednisone +IM (immunosuppressive therapy only)	14 (8.1)	8 (7.7)	6 (8.8)	
Only cholinesterase inhibitors	29 (16.9)	19 (18.3)	10 (14.7)	
Cholinesterase inhibitors + prednisone/IM/prednisone +IM	75 (43.6)	41 (39.4)	34 (50.0)	
Treatment including PE/IG/OT	39 (22.7)	28 (26.9)	11 (16.2)	
Postoperative additional PE + IG + OT treatment	35 (20.4)	26 (25.0)	9 (13.2)	0.061
Achievement of CSR	12 (7.0)	7 (6.7)	5 (7.4)	> 0.99
Achievement of CNR (CSR + PR + MM‐0)	23 (13.4)	12 (11.5)	11 (16.2)	0.38
The favorable outcomes (CSR + PR + MM 0–3)	83 (48.3)	47 (45.2)	36 (52.9)	0.47
Time from ThX to CSR (years)	3.5 (1.8, 5.0)	4.0 (3.0, 7.0)	1.0 (1.0, 3.0)	0.084
Time from ThX to CNR (years)	4.0 (2.0, 5.0)	4.0 (3.5, 8.3)	3.0 (1.5, 4.0)	0.17
5‐year CSR rate (%)	8.3	7.3	9.8	0.59
5‐year CNR rate (%)	14.9	11.2	20.4	0.13
10‐year CSR rate (%)	12.1	12.7	9.8	0.81
10‐year CNR rate (%)	21.0	20.1	20.4	> 0.99
Duration of follow‐up in years (Mean, SD)	5.1 (0.29)	5.3 (0.38)	4.9 (0.45)	0.57

*Note:* Values are presented as median (Interquartile range, IQR) or number (%).

Abbreviations: C‐D, Clavien‐Dindo; CNR, composite neurological remission; CSR, complete stable remission; IG, IVIg therapy; IM, immunosuppression therapy other than prednisone (Azathioprine, Mycophenolate Mofetil, Methotrexate); LND, lymph node dissection; LOMG, late‐onset myasthenia gravis; LOS, length of stay; MG, myasthenia gravis; MM, minimal manifestations; NT, no therapy; OT, other forms of therapy (rituximab, eculizumab, efgartigimod, ravulizumab, daratumumab); PE, plasma exchange therapy; PR, pharmacologic remission; SD, standard deviation; ThX, thymectomy.

^a^ Adverse composite outcomes are defined as having any adverse event: intraoperative conversion, perioperative death (within 30 days and 90 days), readmission, severe postoperative complications, positive margin, and postoperative MG deterioration.

*, Significant at *p* < 0.05; **, Significant at *p* < 0.005. Bolded rows denote clinical factors that showed statistically significant associations (*p* < 0.05) in the multivariable logistic regression analysis. This highlighting aims to distinguish independent predictors identified after adjustment for confounding variables.

Table S1 details postoperative complications. No patient died within 30 or 90 days after surgery. Most complications (83.3%) were minor. There were no significant differences in perioperative parameters and postoperative outcomes, including adverse composite outcomes, between the two groups. Thymic tumors (*p* = 0.042) and combined additional resection (*p* = 0.01) were identified as independent risk factors for adverse composite outcomes (Table S2).

The mean follow‐up time for the entire cohort was 5.1 years (SD: 0.29, 95% CI: 4.57–5.71), with no thymoma‐related recurrences observed. Microscopically positive margins (R1 resection) were identified in one patient from each group (Table 2); both patients were alive and recurrence‐free at the last follow‐up. No R2 resections occurred. In total, five patients died during follow‐up: one from MG‐related causes and four from unrelated conditions, including COVID‐19 and stroke.

**FIGURE 1:**
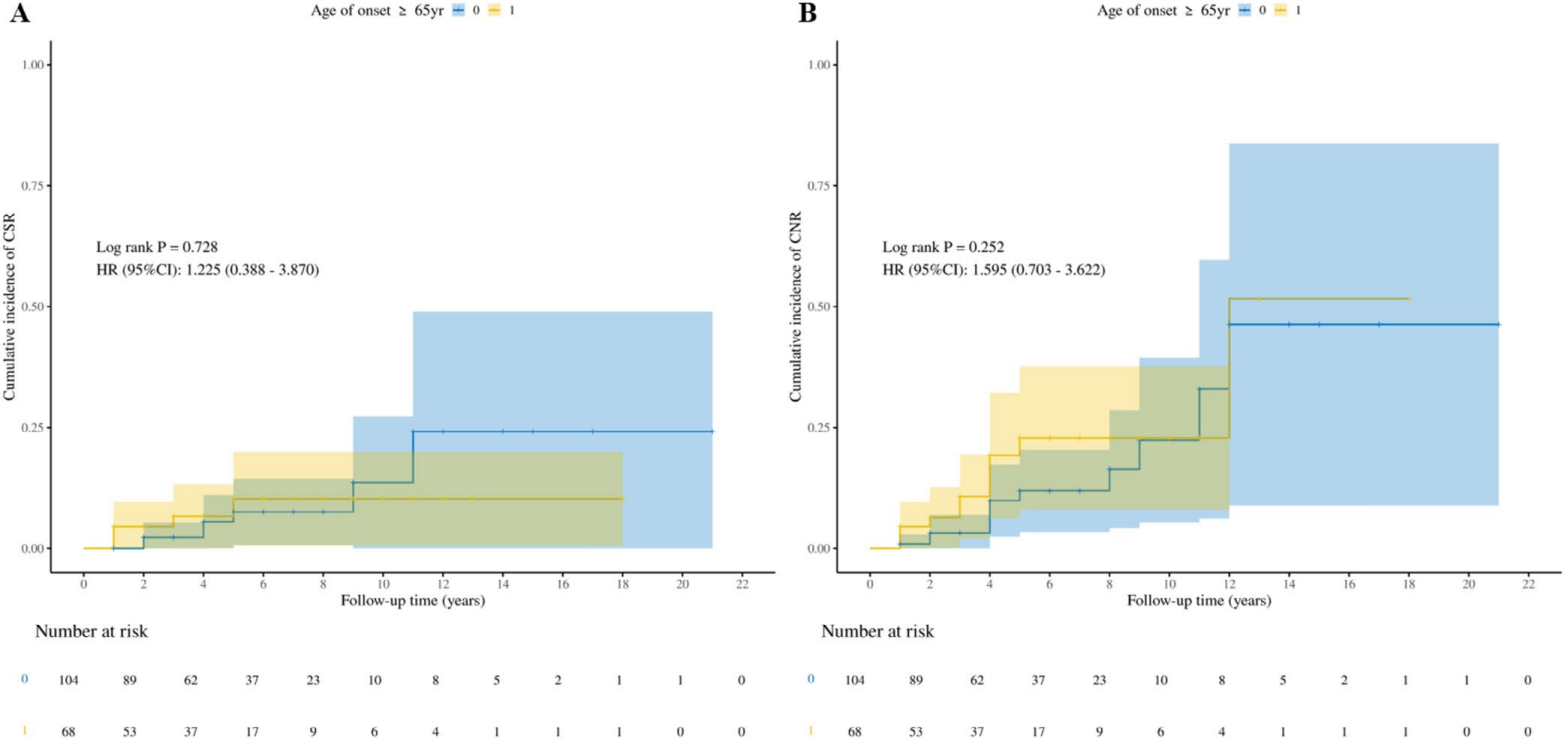
Cumulative incidence of CSR (A) and CNR (B) in the two groups. The blue line and shaded area represent the LOMG group (disease onset at 50–64 years) and its corresponding confidence interval. The yellow line and shaded area represent the V‐LOMG group (disease onset at ≥ 65 years) and its confidence interval. CI, confidence interval; CNR, composite neurological remission (CSR + PR + MM‐0); CSR, complete stable remission; HR, hazard ratio; MM‐0, minimal manifestations‐0; PR, pharmacologic remission.

In the LOMG group, 6.7%, 2.9%, and 1.9% of patients achieved CSR, PR, and MM‐0 status, respectively, while in the V‐LOMG group, the corresponding rates were 7.4%, 5.9%, and 0.9%. The proportions of patients achieving CNR and favorable outcomes were 11.5% and 45.2% in the LOMG group and 16.2% and 52.9% in the V‐LOMG group, with no statistically significant differences in CSR, CNR, or other neurological outcomes between groups (Table 2). Additionally, the median time from surgery to achieving CSR and CNR showed no significant difference. The K‐M curve analysis revealed no differences in the cumulative incidence of CSR (*p* = 0.73, HR: 1.23, 95% CI: 0.39–3.87) or CNR (*p* = 0.25, HR: 1.60, 95% CI: 0.70–3.62) between groups (Figure 1). The 5‐year CSR and CNR rates were 7.3% and 11.2% in the LOMG group and 9.8% and 20.4% in the V‐LOMG group. Similarly, the 10‐year CSR and CNR rates were 12.7% and 20.1% in the LOMG group, and 9.8% and 20.4% in the V‐LOMG group, with no significant intergroup differences (all *p* > 0.1).

**FIGURE 2:**
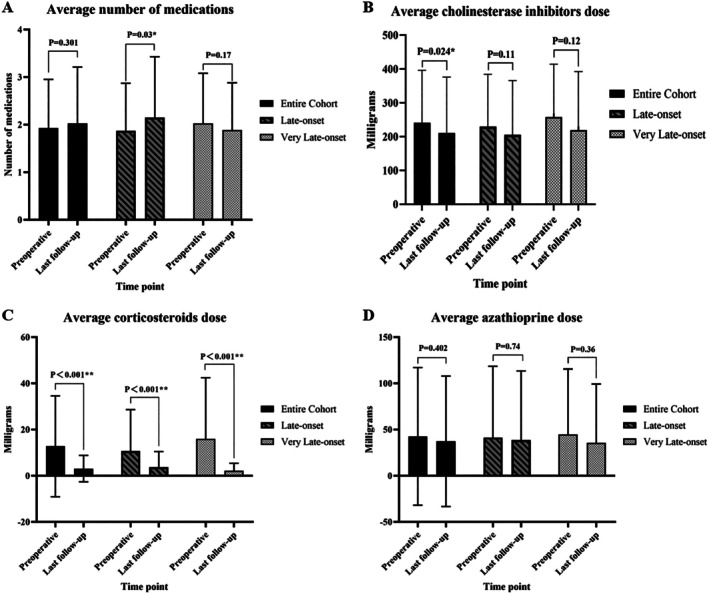
Average daily medication usage before and after robotic extended thymectomy in patients with late‐onset myasthenia gravis. (A) Number of medication types administered preoperatively and at the last follow‐up. (B–D) Average daily dose of (B) cholinesterase inhibitors, (C) corticosteroids, and (D) azathioprine at both time points. Data were obtained from institutional records or structured telephone interviews. Solid bars represent the total cohort; striped bars, patients with onset at 50–64 years (LOMG); and dotted bars, patients with onset ≥ 65 years (V‐LOMG). *p < 0.05, **p < 0.005.

Figure 2 and Table S3 compare average daily medication usage before surgery and at the last follow‐up. Two groups including the entire cohort showed a significant reduction in mean corticosteroid dose at the last follow‐up compared to the preoperative period (both *p* < 0.001), confirming a steroid‐sparing effect, with the V‐LOMG group exhibiting the greatest decrease (16.0 mg vs. 2.1 mg, *p* < 0.001, 95% CI: 7.46 to 20.28). A trend toward decreased mean doses of cholinesterase inhibitors and AZA was observed in both groups and the entire cohort at the last follow‐up. The decrease in cholinesterase inhibitors dose was significant in the entire cohort (from 240.8 mg to 210.8 mg, *p* = 0.024). The LOMG group showed a statistically significant increase in the average number of medication types at the last follow‐up (*p* = 0.03), whereas the V‐LOMG group and the overall cohort demonstrated no significant changes.

In univariable analysis, patients with MGFA class I prior to surgery were significantly more likely to achieve favorable outcomes (*p* = 0.042, OR = 2.10, 95% CI: 1.03–4.40, see Table 3), whereas those with MGFA class III–V showed a trend toward lower remission rates, although without statistical significance. In the multivariable logistic regression model, MGFA classification did not retain independent predictive value.

**TABLE 3:** Multivariable regression analysis of factors associated with favorable outcomes.

Factor	Univariate analysis		Multivariate analysis	
	OR (95% CI)	*p*	OR (95% CI)	*p*
Age of onset ≥ 65 years	1.4 (0.74 ~ 2.52)	0.32		
Male sex	1.7 (0.92 ~ 3.07)	0.092		
With OAID	0.5 (0.25 ~ 1.14)	0.10		
With concomitant disease	0.7 (0.30 ~ 1.47)	0.31		
Positive antibody status	1.4 (0.49 ~ 4.25)	0.51		
GMG at disease onset	0.9 (0.47 ~ 1.56)	0.62		
MGFA classification I before ThX	2.1 (1.03 ~ 4.40)	0.042*	1.3 (0.58 ~ 3.00)	0.50
MGFA classification III‐V before ThX	0.5 (0.18 ~ 1.11)	0.084		
**Preoperative immunosuppressive therapy**	**0.4 (0.18 ~ 0.68)**	0.002**	**0.3 (0.16 ~ 0.73)**	0.006*
Delay from symptom onset to diagnosis ≥ 9 months	0.7 (0.37 ~ 1.43)	0.36		
Delay from onset to ThX ≥ 1 year	0.7 (0.37 ~ 1.24)	0.21		
**Delay from onset to ThX ≥ 2 years**	**0.3 (0.15 ~ 0.69)**	0.004**	**0.4 (0.14 ~ 0.83)**	0.018*
Delay from onset to ThX ≥ 5 years	0.2 (0.02 ~ 1.79)	0.15		
Operation year ≥ 2014	0.5 (0.22 ~ 1.08)	0.077		
Conversion to sternotomy	0 (0.00 ~ Inf)	0.99		
Combined additional resection	0.5 (0.13 ~ 2.15)	0.37		
Thymic tumor	1.9 (1.00 ~ 3.74)	0.051		
Thymic hyperplasia	1.0 (0.47 ~ 2.16)	0.98		
Thymic atrophy	0.7 (0.32 ~ 1.41)	0.29		
Ectopic thymic tissue	0.7 (0.31 ~ 1.76)	0.49		
Postoperative complications	0.4 (0.18 ~ 0.86)	0.019*	0.4 (0.18 ~ 1.02)	0.056
Adverse composite outcomes^a^	0.3 (0.09 ~ 1.28)	0.11		
Postoperative severe complications (C‐D grade ≥ 3)	0.2 (0.02 ~ 1.79)	0.15		
Preoperative MG crisis (impending or manifest)	0.6 (0.23 ~ 1.44)	0.24		
Postoperative impending MG crisis or manifest crisis (≤ 1 month)	0.4 (0.12 ~ 1.33)	0.14		
Postoperative impending MG crisis or manifest crisis (> 1 month)	0.3 (0.11 ~ 0.91)	0.033*	0.4 (0.13 ~ 1.50)	0.19
**Postoperative additional PE + IG + OT treatment**	**0.1 (0.05 ~ 0.34)**	< 0 0.001**	**0.2 (0.06 ~ 0.51)**	0.001**

Abbreviations: C‐D, Clavien‐Dindo; CI, confidence interval; GMG, generalized myasthenia gravis; IG, IVIg therapy; MG, myasthenia gravis; MGFA, Myasthenia Gravis Foundation of America; OAID, other auto‐immune diseases; OR, odds ratio; OT, other forms of therapy (rituximab, eculizumab, efgartigimod, ravulizumab, daratumumab); PE, plasma exchange therapy; ThX, thymectomy.

^a^ Adverse composite outcomes are defined as having any adverse event: intraoperative conversion, perioperative death (within 30 days and 90 days), readmission, severe postoperative complications, positive margin, and postoperative MG deterioration.

*, *p* < 0.05 with a statistical difference; **, *p* < 0.005 with a statistical difference. Bolded rows denote clinical factors that showed statistically significant associations (*p* < 0.05) in the multivariable logistic regression analysis. This highlighting aims to distinguish independent predictors identified after adjustment for confounding variables.

Preoperative immunosuppressive therapy (*p* = 0.006), thymectomy delay over 2 years (*p* = 0.018), and postoperative additional PE + IG + OT treatment (*p* = 0.001) were identified as independent risk factors negatively affecting the likelihood of achieving favorable outcomes (Table 3). Moreover, preoperative immunosuppressive therapy was the only clinical factor independently linked to a significantly lower probability of achieving CSR (*p* = 0.042) and CNR (*p* = 0.018) (Table S4), further underscoring its predictive role in unfavorable outcomes.

## 4 Discussion

This 21‐year single‐center retrospective study presents a large cohort of LOMG patients who underwent RATS extended thymectomy and utilizes composite measures to systematically assess short‐term postoperative and long‐term neurological outcomes. The findings support a personalized treatment approach, including surgical intervention, for carefully selected patients likely to benefit, with a mean follow‐up of 5.1 years.

The age criteria for LOMG vary across studies, as detailed in Table S5. While some studies set the cutoff at 40–45 years [10, 11], most literature [12, 13, 14, 15, 16, 17, 18] defines 50 years as the boundary between LOMG and EOMG, with 65 years and above classified as V‐LOMG [19, 20]. However, the heterogeneous definitions of age may be based on onset age, enrollment age, diagnosis age, or treatment age. Notably, 27% of patients reportedly waited over a year for an accurate diagnosis [21], suggesting that some EOMG cases in other studies [15, 18] may have been misclassified as LOMG. This study used onset age as the grouping criterion, minimizing the impact of diagnostic and treatment delays.

This study found that V‐LOMG patients experienced shorter intervals from symptom onset to diagnosis and thymectomy, with significantly fewer cases exhibiting a thymectomy delay of ≥ 1 or ≥ 2 years. However, these clinical factors were not significantly associated with CSR and CNR achievement. While no significant association was observed between diagnostic delay and postoperative outcomes in this study, it remains a clinically relevant factor, as delayed diagnosis may contribute to thymoma progression, an increase in MGFA classification [21], and deterioration in quality of life [21] due to prolonged fatigue, psychological distress, and delayed symptom control.

Notably, a thymectomy delay exceeding 2 years was identified as an independent adverse prognostic factor for favorable outcomes. Studies by Chung et al. and Cunha et al. reported that an onset‐to‐surgery interval of less than 2 years [28] and less than 1 year [29], respectively, was associated with better outcomes. Collectively, these findings, along with the present study, emphasize the importance of early MG diagnosis and timely surgical intervention in optimizing patient prognosis.

Although preoperative MGFA classification is widely recognized as a potential predictor of neurological outcomes after thymectomy, it was not identified as an independent prognostic factor in this multivariable analysis. This finding contrasts with studies such as that by Marulli et al. [30], which demonstrated higher remission rates in patients with MGFA class I–II. However, their cohort primarily comprised younger patients with EOMG. In our late‐onset population, age‐related comorbidities and clinical heterogeneity may attenuate the predictive value of MGFA classification. Moreover, a previous study [31] suggests that remission in patients with higher MGFA classes may be time‐dependent, becoming more apparent over extended follow‐up periods. Therefore, the mean follow‐up duration of 5.1 years may not have been sufficient to fully explore the prognostic significance of MGFA classification in this late‐onset subgroup.

The year 2014 marked the transition in this center from the first‐generation da Vinci system to the Si platform, which introduced several key technological advancements and enabled the adoption of novel approaches such as the subxiphoid four‐trocar technique. Therefore, 2014 was selected as a milestone to distinguish between early and late surgical experience. In this study, no significant differences in postoperative neurological outcomes were observed between the two time periods. This may reflect that surgical techniques, resection strategies, and perioperative management had already been standardized before this transition. While Comacchio et al. [32] reported a progressive improvement in outcomes over time by analyzing the year of surgery as a continuous variable, this study employed a categorical time‐point approach and focused specifically on patients with late‐onset MG, a subgroup in whom the long‐term neurological benefit from surgical and technological advancements may be less pronounced.

Thymoma and combined additional resection were identified as independent risk factors for adverse composite outcomes in this study. However, thymoma was not an independent negative predictor for achieving CSR or CNR. Previous studies reported more frequent and severe postoperative complications in thymoma‐associated MG compared to nonthymomatous MG [33]. Therefore, for MG patients with suspected thymoma, minimally invasive thymectomy can be considered if R0 resection is ensured, nerve injury is minimized, and unnecessary resection is avoided to reduce complications and improve long‐term neurological outcomes.

In this study, the overall prevalence of thymic follicular hyperplasia and thymic atrophy was 19.2% and 21.5%, respectively. Most studies report thymic hyperplasia rates in LOMG ranging from 5% to 30% [16, 20, 25]. However, thymic atrophy and hyperplasia did not emerge as significant independent predictors of neurological outcomes. Uzawa et al. [14] observed that among nonthymomatous LOMG patients, those with hyperplastic thymus derived greater benefit from thymectomy, achieving higher remission rates and requiring lower daily doses of prednisone. In contrast, a retrospective subgroup analysis [15] found no association between follicular hyperplasia and the achievement of neurological outcomes (PR/CSR) in elderly NTMG patients, suggesting that the prognostic role of hyperplasia may vary across patient populations and study designs. However, in our LOMG cohort, the incidence of follicular hyperplasia was relatively low, which may have limited its predictive value in the multivariable analysis.

Regarding thymic atrophy, our findings are consistent with those of Chen et al. [34], who reported no significant differences in postoperative prognosis between patients with thymic atrophy and those with hyperplasia. In contrast, Nakahara et al. [35] reported a retrospective study involving 93 MG patients, in which those with thymic atrophy experienced the most stable postoperative course and the most favorable clinical prognosis. These inconsistencies across studies may reflect variations in patient populations, definitions of atrophy, and follow‐up durations, highlighting the need for further large‐scale, long‐term research in this area, with a particular focus on the LOMG population.

Moreover, LOMG patients demonstrated a significant steroid‐sparing effect, with a more pronounced reduction in the V‐LOMG group, consistent with previous findings [12]. Preoperative immunosuppressive therapy and postoperative PE + IG + OT treatment were identified as independent adverse predictors for achieving favorable neurological outcomes in this study. Patients requiring both preoperative immunosuppressive therapy and postoperative PE + IG + OT treatment likely represent a more severe disease course, requiring intensive management and potentially experiencing poorer neurological outcomes regardless of thymectomy.

The complication rate reported in this study falls within the range of 5.5% [25] to 32.1% [22] reported in other studies on thymectomy in LOMG patients. Compared to studies on EOMG cohorts undergoing thymectomy [12, 25], this study found no evidence of harm associated with thymectomy in LOMG patients. The reported association between thymectomy and increased all‐cause mortality or cancer risk by Kooshesh et al. [36] was not supported by the follow‐up data in this study. Furthermore, their findings do not negate the role of thymectomy when a clear surgical indication, such as thymoma and/or MG, is present [37].

A subgroup analysis [38] from a systematic review and meta‐analysis by Chen et al. reported that thymectomy appeared to be more effective than medical therapy alone in patients with MG onset at ≥ 45 years or those who underwent thymectomy at ≥ 50 years of age. It is essential to recognize that thymectomy is not required for all LOMG patients. With advancements in novel pharmacological therapies for MG, timely medical interventions that prevent disease progression and achieve complete control without surgery are equally accepted by surgeons.

The study was conducted by a dedicated MG treatment team, including thoracic surgeons and neurologists, ensuring standardized surgical indications, methods, and treatment strategies, thereby minimizing heterogeneity in data collection and analysis.

However, as this was a single‐center retrospective study, selection bias may exist. Furthermore, although MGFA‐PIS was used to assess neurological outcomes, other quantifiable MG severity metrics were not included. In addition, adverse effects of immunosuppressive therapies were not recorded, limiting the evaluation of treatment tolerability. Moreover, longer follow‐up is needed to confirm the durability of neurological improvement. Finally, due to the small number of patients receiving novel therapies, dosage comparisons could not be performed.

## 5 Conclusion

RATS extended thymectomy is a safe and feasible option for patients with MG onset at age ≥ 50 years, including those with V‐LOMG, providing favorable neurological outcomes with a significant steroid‐sparing effect.

## Author Contributions

**Luyu Huang:** conceptualization, methodology, data curation, formal analysis, investigation, writing – review and editing, writing – original draft, validation, visualization. **Feng Li:** conceptualization, data curation, methodology, investigation, funding acquisition, writing – original draft, writing – review and editing. **Zhongmin Li:** data curation, investigation, resources. **Hongbin Zhang:** data curation, resources, investigation. **Aron Elsner:** resources, visualization. **Julia Strauchmann:** resources, visualization. **Marco Nicolas Andreas:** resources, visualization. **Tomasz Dziodzio:** resources, visualization. **Aina Lask:** resources, visualization. **Jens Neudecker:** resources, visualization. **Daipeng Xie:** methodology, software, data curation. **Lintong Yao:** methodology, software. **Shaowei Wu:** methodology, software. **Haiyu Zhou:** methodology, project administration, writing – original draft, writing – review and editing. **Andreas Meisel:** conceptualization, supervision, methodology, funding acquisition, project administration, writing – original draft, writing – review and editing. **Jens‐C. Rueckert:** conceptualization, methodology, project administration, supervision, writing – original draft, writing – review and editing.

## Conflicts of Interest

Proctors for Intuitive Surgical (Jens‐C. Rueckert and Aron Elsner). Member of the medical advisory board of the German MG Society (Andreas Meisel, Jens‐C. Rueckert). Andreas Meisel received speaker or consultancy honoraria or financial research support (paid to his institution) from Alexion Pharmaceuticals, Amgen, argenx, Axunio, Desitin, Grifols, Hormosan Pharma, Johnson and Johnson, Merck, Novartis, Octapharma, Sanofi, and UCB.

## Supporting information


**FIGURE S1:** Flow diagram showing patient selection in the study. MG, myasthenia gravis; VATS, video‐assisted thoracic surgery.

**FIGURE S2:** The trends in RATS extended thymectomy for LOMG over time in this center and the distribution of MGFA clinical classification between the two subgroups defined by disease onset age. In both panels, blue bars represent patients with disease onset at 50–64 years (LOMG group), while orange bars represent those with disease onset at ≥ 65 years (V‐LOMG group). (A) Trends in RATS extended thymectomy for LOMG at Charité Universitätsmedizin Berlin from 2003 to 2023, showing the number of patients who underwent surgery in different time periods. (B) Distribution of MGFA clinical classification between the LOMG and V‐LOMG groups, depicting the number of patients in each MGFA class. RATS, robotic‐assisted thoracic surgery; LOMG, late‐onset myasthenia gravis; MGFA, Myasthenia Gravis Foundation of America; V‐LOMG, very late‐onset myasthenia gravis.

**TABLE S1:** Postoperative complications stratified by the age of MG onset in cohorts.

**TABLE S2:** Univariate and multivariate analysis for predictors of adverse composite outcomes in LOMG patients.

**TABLE S3:** Average medication doses of the late‐onset and very late‐onset groups.

**TABLE S4:** Multivariable regression analysis of factors associated with CSR and CNR.

**TABLE S5:** Summary of major studies on thymectomy in the LOMG patients.

**Data S1:** ene70388‐sup‐0008‐Supinfo.docx.

## Data Availability

The datasets generated and/or analyzed during the current study are available from the corresponding author.
