# Supplementary figures and images for: Robotic Extended Thymectomy in Late‐Onset Myasthenia Gravis: A 21‐Year Retrospective Cohort Study of 172 Patients
Source: Eur J Neurol. 2025 Nov 5;32(11):e70388. doi: 10.1111/ene.70388 (PMC12587165; doi:10.1111/ene.70388)

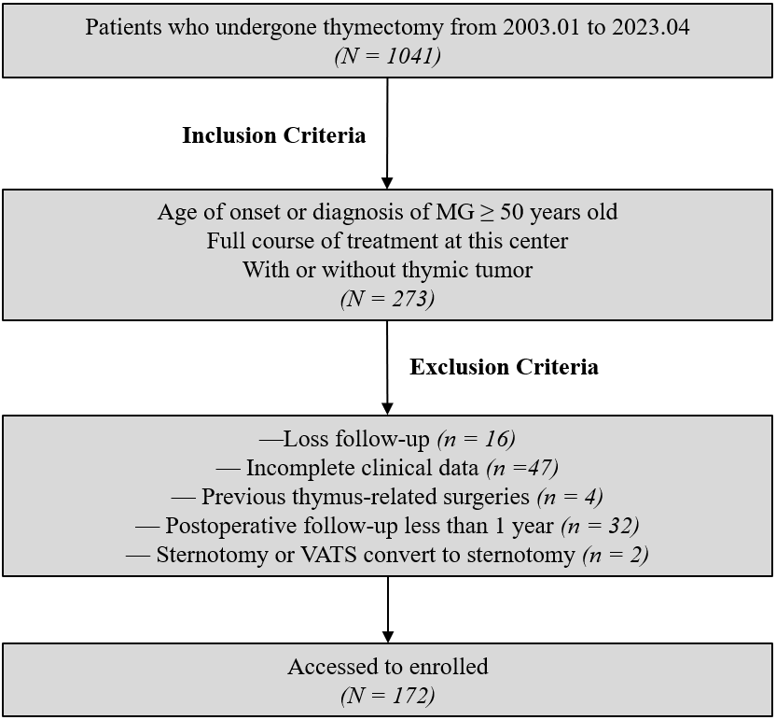

Supplement: Supplementary file 1 — FIGURE S1: Flow diagram showing patient selection in the study. MG, myasthenia gravis; VATS, video‐assisted thoracic surgery. [file ENE-32-e70388-s007.tif]

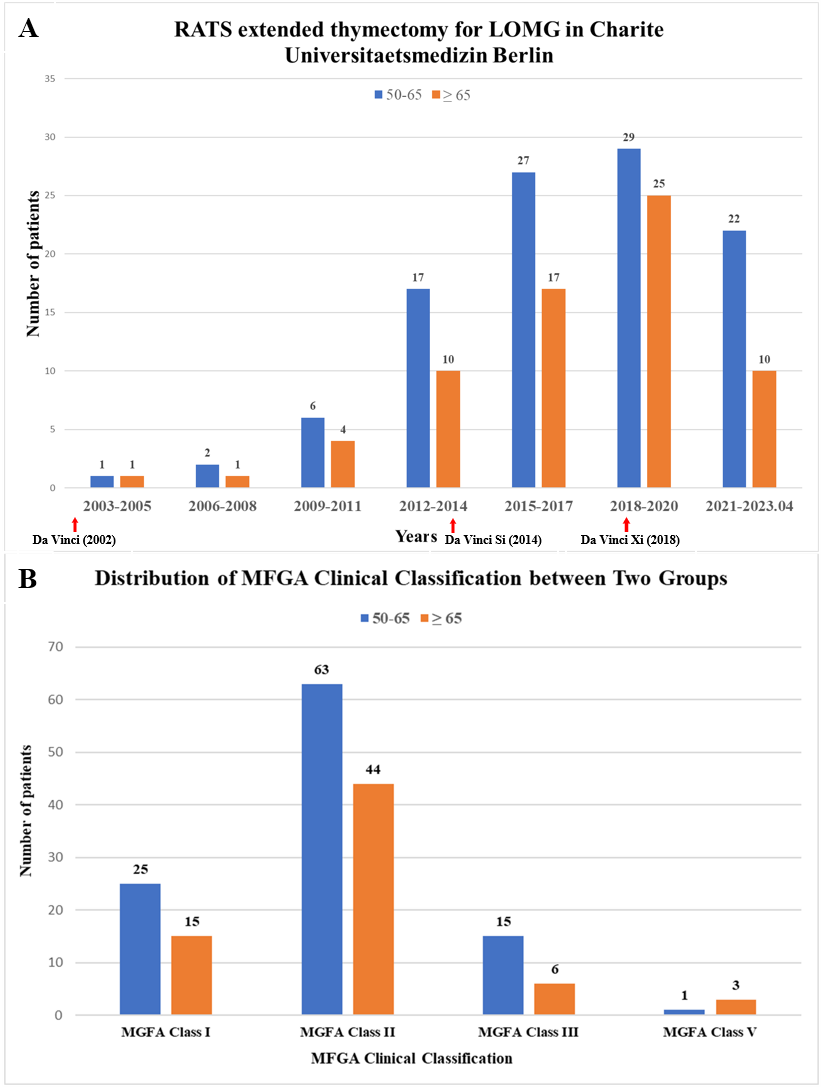

Supplement: Supplementary file 2 — FIGURE S2: The trends in RATS extended thymectomy for LOMG over time in this center and the distribution of MGFA clinical classification between the two subgroups defined by disease onset age. In both panels, blue bars represent patients with disease onset at 50–64 years (LOMG group), while orange bars represent those with disease onset at ≥ 65 years (V‐LOMG group). (A) Trends in RATS extended thymectomy for LOMG at Charité Universitätsmedizin Berlin from 2003 to 2023, showing the number of patients who underwent surgery in different time periods. (B) Distribution of MGFA clinical classification between the LOMG and V‐LOMG groups, depicting the number of patients in each MGFA class. RATS, robotic‐assisted thoracic surgery; LOMG, late‐onset myasthenia gravis; MGFA, Myasthenia Gravis Foundation of America; V‐LOMG, very late‐onset myasthenia gravis. [file ENE-32-e70388-s003.tif]
