# Supplementary material for: Robotic Extended Thymectomy in Late‐Onset Myasthenia Gravis: A 21‐Year Retrospective Cohort Study of 172 Patients
Source: Eur J Neurol. 2025 Nov 5;32(11):e70388. doi: 10.1111/ene.70388 (PMC12587165; doi:10.1111/ene.70388)
Supplement: Supplementary file 3 — TABLE S1: Postoperative complications stratified by the age of MG onset in cohorts. [file ENE-32-e70388-s004.docx]

**Supplemental Table 1. Postoperative Complications Stratified by The Age of MG Onset in Cohorts**

| **Characteristics** | **Entire Cohort** | **Late-onset MG (≥ 50 to < 65)** | **Very-late-onset MG (**≥ **65)** | ***P*** |
| --- | --- | --- | --- | --- |
|  | **N =172** | **n = 104** | **n = 68** |  |
| **Postoperative C-D complication grade, No. (%) ^a^** |  |  |  | 0.18 |
| Grade 0 | 136 (79.1) | 84 (80.8) | 52 (76.5) |  |
| Grade 1 | 13 (7.6) | 7 (6.7) | 6 (8.8) |  |
| Postoperative diaphragm elevation | 4 | 3 | 1 |  |
| Pleural effusion | 1 | 0 | 1 |  |
| Subcutaneous hematoma | 1 | 0 | 1 |  |
| Pneumothorax | 3 | 2 | 1 |  |
| Anesthesia complications | 4 | 2 | 2 |  |
| Grade 2 | 17 (9.9) | 12 (11.5) | 5 (7.4) |  |
| Myasthenic symptoms deterioration | 11 | 9 | 2 |  |
| Cardiac arrhythmia | 5 | 2 | 3 |  |
| Renal insufficiency | 1 | 1 | 0 |  |
| Grade 3 | 1 (0.6) | 0 (0) | 1 (1.5) |  |
| Pleural effusion requiring chest tube placement | 1 | 0 | 1 |  |
| Grade 4 | 5 (2.9) | 1 (1.0) | 4 (5.9) |  |
| Pulmonary embolism | 2 | 0 | 2 |  |
| MG crisis | 2 | 1 | 1 |  |
| Acute renal failure | 1 | 0 | 1 |  |
| Grade 5 | 0 (0) | 0 (0) | 0 (0) |  |
| Values are presented as number (%). C-D, Clavien-Dindo; MG, Myasthenia gravis; *, Significant at p＜0.05; **, Significant at p＜0.005. Values are presented as mean± standard deviation/median (IQR) or number (%).^a^ Clavien-Dindo grade, I: Any deviation from the normal postoperative course without the need for pharmacological treatment or surgical, endoscopic, and radiological interventions; allowed regimens such as antiemetics, antipyretics, analgesics, diuretics, electrolytes, and physiotherapy; II: Requiring pharmacological treatment with drugs other than such allowed for grade I complications; blood transfusions and total parenteral nutrition are also included; III: Requiring surgical, endoscopic or radiological intervention; IV: Life-threatening complication requiring intermediate care/intensive care unit management; V: Death of a patient. | | | | |
