# Supplementary material for: Robotic Extended Thymectomy in Late‐Onset Myasthenia Gravis: A 21‐Year Retrospective Cohort Study of 172 Patients
Source: Eur J Neurol. 2025 Nov 5;32(11):e70388. doi: 10.1111/ene.70388 (PMC12587165; doi:10.1111/ene.70388)
Supplement: Supplementary file 4 — TABLE S2: Univariate and multivariate analysis for predictors of adverse composite outcomes in LOMG patients. [file ENE-32-e70388-s002.docx]

**Supplemental Table 2. Univariate and Multivariate Analysis for Predictors of Adverse Composite Outcomes ^a^ in LOMG Patients**

| **Variables** | **Univariate analysis** | | **Multivariate analysis** | |
| --- | --- | --- | --- | --- |
|  | **OR (95% CI)** | ***P*** | **OR (95% CI)** | ***P*** |
| Age of onset ≥ 65yr | 2.27 (0.69 ~ 7.48) | 0.18 |  |  |
| Male sex | 0.45 (0.13 ~ 1.56) | 0.21 |  |  |
| With OAID | 1.28 (0.33 ~ 5.01) | 0.72 |  |  |
| With concomitant disease | 29018400.9 (0.0 ~ Inf) | 0.99 |  |  |
| Positive antibody status | 9570934.6 (0.0 ~ Inf) | 0.99 |  |  |
| GMG at disease onset | 1.22 (0.38 ~ 3.95) | 0.74 |  |  |
| MGFA classification I before ThX | 1.11 (0.29 ~ 4.31) | 0.88 |  |  |
| MGFA classification III-V before ThX | 0.0 (0.0 ~ Inf) | 0.99 |  |  |
| Preoperative immunosuppressive therapy | 0.88 (0.25 ~ 3.07) | 0.85 |  |  |
| Preoperative MG crisis (impending or manifest) | 0.60 (0.07 ~ 4.90) | 0.64 |  |  |
| Delay from symptom onset to diagnosis ≥ 9 months | 0.88 (0.23 ~ 3.40) | 0.85 |  |  |
| Delay from onset to ThX ≥ 1 year | 0.57 (0.16 ~ 1.96) | 0.37 |  |  |
| Delay from onset to ThX ≥ 2 years | 0.28 (0.04 ~ 2.25) | 0.23 |  |  |
| Delay from onset to ThX ≥ 5 years | 2.82 (0.30 ~ 26.27) | 0.36 |  |  |
| Operation year ≥ 2014 | 1.15 (0.24 ~ 5.54) | 0.86 |  |  |
| Thymic hyperplasia | 0.83 (0.17 ~ 3.99) | 0.82 |  |  |
| Thymic atrophy | 1.92 (0.55 ~ 6.78) | 0.31 |  |  |
| Ectopic thymic tissue | 0.54 (0.07 ~ 4.39) | 0.57 |  |  |
| **Thymic tumor** | **8.16 (2.11 ~ 31.57)** | **0.002**** | **5.23 (1.06 ~ 25.86)** | **0.042*** |
| **Combined additional resection** | **27.86 (6.11 ~ 127.02)** | **< 0.001**** | **18.8 (2.04 ~ 173.09)** | **0.01*** |
| CI, Confidence interval; GMG, Generalized myasthenia gravis; LOMG, Late-onset myasthenia gravis; MG, Myasthenia gravis; MGFA, Myasthenia Gravis Foundation of America; OAID, Other auto-immune diseases; OR, Odds ratio; ThX, Thymectomy; *, Significant at p＜0.05; **, Significant at p＜0.005.  a Adverse composite outcomes are defined as having any adverse event: intraoperative conversion, perioperative death (within 30 days and 90 days), readmission, severe postoperative complications, positive margin, and postoperative MG deterioration. | | | | |
