# Supplementary material for: Robotic Extended Thymectomy in Late‐Onset Myasthenia Gravis: A 21‐Year Retrospective Cohort Study of 172 Patients
Source: Eur J Neurol. 2025 Nov 5;32(11):e70388. doi: 10.1111/ene.70388 (PMC12587165; doi:10.1111/ene.70388)
Supplement: Supplementary file 5 — TABLE S3: Average medication doses of the late‐onset and very late‐onset groups. [file ENE-32-e70388-s001.docx]

**Supplemental Table 3. Average Medication Doses of The Late-Onset and Very Late-Onset Groups**

| **Medication** | **Preoperative** | **Last follow-up** | ***P*** | **95% CI** |
| --- | --- | --- | --- | --- |
| Number of medication types, No. |  |  |  |  |
| Entire Cohort | 1.9 (1.0) | 2.0 (1.2) | 0.30 | -0.29 to 0.089 |
| **Late-onset (50-65)** | **1.9 (****1.0)** | **2.2 (****1.3)** | **0.03*** | **-0.54 to -0.04** |
| Very Late-onset (≥ 65) | 2.0 (1.1) | 1.8 (0.99) | 0.17 | -0.08 to 0.46 |
| Cholinesterase inhibitors (mg), mean (SD) |  |  |  |  |
| **Entire Cohort** | **240.8 (****155.1)** | **210.8 (165.2)** | **0.024*** | **4.09 to 56.09** |
| Late-onset (50-65) | 230.0 (154.0) | 205.3 (160.3) | 0.11 | -5.27 to 54.70 |
| Very Late-onset (≥ 65) | 257.4 (156.4) | 219.1 (173.2) | 0.12 | -9.80 to 86.41 |
| Prednisone (mg), mean (SD) |  |  |  |  |
| **Entire Cohort** | **12.8 (****21.8)** | **3.1 (****5.7)** | **＜0.001**** | **6.44 to 12.92** |
| **Late-onset (50-65)** | **10.7 (****18.0)** | **3.7 (****6.8)** | **＜0.001**** | **3.61 to 10.27** |
| **Very Late-onset (≥ 65)** | **16.0 (****26.5)** | **2.1 (****3.3)** | **＜0.001**** | **7.46 to 20.28** |
| Azathioprine (mg), mean (SD) |  |  |  |  |
| Entire Cohort | 42.7 (74.5) | 37.2 (70.6) | 0.40 | -7.42 to 18.41 |
| Late-onset (50-65) | 41.3 (77.2) | 38.5 (74.9) | 0.74 | -13.93 to 19.60 |
| Very Late-onset (≥ 65) | 44.9 (70.7) | 35.3 (64.0) | 0.36 | -11.16 to 30.29 |
| Values are presented as mean ± standard deviation (SD) or number (%). CI, Confidence interval; SD, Standard deviation;*, Significant at p＜0.05; **, Significant at p＜0.005. | | | | |
