# Supplementary material for: Robotic Extended Thymectomy in Late‐Onset Myasthenia Gravis: A 21‐Year Retrospective Cohort Study of 172 Patients
Source: Eur J Neurol. 2025 Nov 5;32(11):e70388. doi: 10.1111/ene.70388 (PMC12587165; doi:10.1111/ene.70388)
Supplement: Supplementary file 6 — TABLE S4: Multivariable regression analysis of factors associated with CSR and CNR. [file ENE-32-e70388-s006.docx]

**Supplemental Table 4. Multivariable Regression Analysis of Factors Associated with CSR and CNR**

| **Factor** | **Complete stable remission** | | **Composite neurological remission (CSR+PR+MM0)** | |
| --- | --- | --- | --- | --- |
|  | **HR (95% CI)** | ***P*** | **HR (95% CI)** | ***P*** |
| Age of onset ≥ 65yr | 1.10 (0.33 ~ 3.62) | 0.88 | 1.48 (0.61 ~ 3.58) | 0.38 |
| Male sex | 1.37 (0.42 ~ 4.48) | 0.61 | 1.28 (0.53 ~ 3.11) | 0.58 |
| With OAID | 0.74 (0.16 ~ 3.54) | 0.71 | 0.77 (0.24 ~ 2.42) | 0.65 |
| With concomitant disease | 0.39 (0.11 ~ 1.38) | 0.14 | 0.54 (0.19 ~ 1.52) | 0.25 |
| Positive antibody status | 9570934.5 (0.0 ~ Inf) | 0.99 | 0.58 (0.15 ~ 2.25) | 0.43 |
| GMG at disease onset | 0.58 (0.17 ~ 2.01) | 0.39 | 0.92 (0.38 ~ 2.22) | 0.85 |
| MGFA classification I before ThX | 2.55 (0.76 ~ 8.53) | 0.13 | 1.95 (0.76 ~ 5.01) | 0.17 |
| MGFA classification III-V before ThX | 0.52 (0.06 ~ 4.17) | 0.53 | 0.24 (0.03 ~ 1.84) | 0.17 |
| **Preoperative immunosuppressive therapy** | **0.29 (0.09 ~ 0.95)** | **0.042*** | **0.33 (0.13 ~ 0.83)** | **0.018*** |
| Delay from symptom onset to diagnosis ≥ 9 months | 0.51 (0.11 ~ 2.42) | 0.39 | 0.71 (0.25 ~ 2.03) | 0.52 |
| Delay from onset to ThX ≥ 1 year | 0.37 (0.10 ~ 1.41) | 0.15 | 0.47 (0.18 ~ 1.20) | 0.11 |
| Delay from onset to ThX ≥ 2 years | 0.64 (0.13 ~ 3.06) | 0.58 | 0.45 (0.13 ~ 1.62) | 0.22 |
| Delay from onset to ThX ≥ 5 years | 0.0 (0.0 ~ Inf) | 0.99 | 0.0 (0.0 ~ Inf) | 0.99 |
| Operation year ≥ 2014 | 0.28 (0.08 ~ 0.96) | 0.06 | 0.48 (0.17 ~ 1.31) | 0.15 |
| Conversion to sternotomy | 0.0 (0.0 ~ Inf) | 0.99 | 0.0 (0.0 ~ Inf) | 0.99 |
| Combined additional resection | 0.0 (0.0 ~ Inf) | 0.99 | 0.80 (0.10 ~ 6.72) | 0.84 |
| Thymic tumor | 1.72 (0.52 ~ 5.68) | 0.38 | 1.96 (0.80 ~ 4.81) | 0.14 |
| Thymic hyperplasia | 0.36 (0.05 ~ 2.92) | 0.34 | 0.60 (0.17 ~ 2.13) | 0.43 |
| Thymic atrophy | 1.92 (0.55 ~ 6.78) | 0.31 | 1.34 (0.49 ~ 3.69) | 0.57 |
| Ectopic thymic tissue | 0.54 (0.07 ~ 4.39) | 0.57 | 0.25 (0.03 ~ 1.94) | 0.18 |
| Postoperative complications | 1.28 (0.33 ~ 5.01) | 0.72 | 1.40 (0.51 ~ 3.86) | 0.52 |
| Postoperative severe complications (C-D grade ≥ 3) | 2.82 (0.30 ~ 26.27) | 0.36 | 1.31 (0.15 ~ 11.74) | 0.81 |
| Adverse composite outcomes ^a^ | 1.23 (0.15 ~ 10.43) | 0.85 | 1.32 (0.27 ~ 6.47) | 0.73 |
| Preoperative MG crisis (impending or manifest) | 0.0 (0.0 ~ Inf) | 0.99 | 0.61 (0.13 ~ 2.82) | 0.53 |
| Postoperative impending MG crisis or manifest crisis (≤ 1 month) | 1.03 (0.12 ~ 8.60) | 0.98 | 1.88 (0.48 ~ 7.33) | 0.36 |
| Postoperative impending MG crisis or manifest crisis (> 1 month) | 0.0 (0.0 ~ Inf) | 0.99 | 0.31 (0.04 ~ 2.44) | 0.27 |
| Postoperative additional PE+IG+OT treatment | 0.0 (0.0 ~ Inf) | 0.99 | 0.33 (0.07 ~ 1.50) | 0.15 |
| C-D, Clavien-Dindo; CI, Confidence interval; CNR, Composite neurological remission; CSR, Complete stable remission; HR, Hazard ratio; GMG, Generalized myasthenia gravis; IG, IVIg therapy; LOMG, Late-onset myasthenia gravis; MG, Myasthenia gravis; MGFA, Myasthenia Gravis Foundation of America; MM, Minimal manifestations; OAID, Other auto-immune diseases; OT, Other forms of therapy (rituximab, eculizumab, efgartigimod, ravulizumab, daratumumab); PE, Plasma exchange therapy; PR, Pharmacologic remission; ThX, Thymectomy; *, *p* < 0.05 with a statistical difference; **, *p* < 0.005 with a statistical difference.  ^a^ Adverse composite outcomes are defined as having any adverse event: intraoperative conversion, perioperative death (within 30 days and 90 days), readmission, severe postoperative complications, positive margin, and postoperative MG deterioration. | | | | |
