# Supplementary material for: Robotic Extended Thymectomy in Late‐Onset Myasthenia Gravis: A 21‐Year Retrospective Cohort Study of 172 Patients
Source: Eur J Neurol. 2025 Nov 5;32(11):e70388. doi: 10.1111/ene.70388 (PMC12587165; doi:10.1111/ene.70388)
Supplement: Supplementary file 7 — TABLE S5: Summary of major studies on thymectomy in the LOMG patients. [file ENE-32-e70388-s008.docx]

**Supplemental Table 5. Summary of major studies on thymectomy in the LOMG patients**

| **Authors** | **Country** | **Study interval** | **ThX in LOMG** | **LOMG** | **Age**  **Cut-off** | **Hyperplasia (%)** | **Thymoma (%)** | **AChR-Ab (+) (%)** | **Male (%)** | **FU**  **(mo)** | **Response rate (%) in ThX LOMG** ^a^ | **Response rate (%) in ThX EOMG** ^a^ |
| --- | --- | --- | --- | --- | --- | --- | --- | --- | --- | --- | --- | --- |
| Present series | Germany(S) | 2003-2024 | 172 | 172 | 50, 65  (Onset) | 12.8% | 29.7% | 91.28% | 51.2% | 61 | 48.3% (CSR+PR+MM0-3),  77.9%(Improved) | NA |
| Tang and colleagues [19] | China (S) | 2015-2021 | 145 | 562 | 50, 65  (Onset) | 13.0% | 19.6% | 55.0% | 54.5% | 40 | NA | NA |
| Jiao and colleagues [22] | China (S) | 2011-2022 | 84 | 84 | 65  (Age) | NA | 33.3% | NA | 63.1% | NA | NA | NA |
| Previous series [23] | Germany(S) | 2003-2017 | 68 | 68 | 60  (Onset) | 17.6% | 36.8% | 95.6% | 63.2% | 60 | 14.7% (CSR+PR+MM-0) | NA |
| Lococo and colleagues [24] | Italy (B) | 2000-2022 | 66 | 66 | 65  (Age) | 0.0% | 100.0% | 78.8% | 34.8% | NA | 22.8% (CSR+PR) | NA |
| Uzawa and colleagues [14] | Japan (S) | 2008-2014 | 39 | 39 | 50  (Onset) | 12.8% | 0.0% | 100.0% | 51.3% | 24 | 30.8%(CSR+PR) | NA |
| Kim and colleagues [15] | South Korea (S) | 1990-2018 | 34 | 139 | 50  (Age) | 52.9% | 0.0% | 100.0% | 23.5% | 75.5 | 17.6% (CSR) | NA |
| Menghesha and colleagues [12] | Germany (S) | 2012-2020 | 28 | 28 | 50  (Onset) | NA | 0.0% | 92.9% | 57.1% | 46 | 61.9% (Improved) | 51.6%(Improved) |
| Abt and colleagues [25] | United States (S) | 1980-1998 | 28 | 28 | 55  (Age) | 28.6% | 10.7% | NA | 71.4% | 58.5 | 10.7% (CSR) | 15.6%(CSR) no difference |
| Tsuchida and colleagues [26] | Japan (S) | 1985-1996 | 25 | 25 | 60  (Age) | 16.0% | 40.0% | 100.0% | 40.0% | 52.8 | 8% (CSR) | 40%(CSR) |
| Sakai and colleagues [20] | Japan (S) | 1978-2014 | 22 | 58 | 50,65  (Onset) | 5.2% | 20.7% | 87.9% | 44.8% | NA | NA | NA |
| Romi and colleagues [13] | Norway (S) | 1969-1999 | 21 | 43 | 50  (Onset) | NA | 0.0% | 100.0% | 52.4% | > 24 | 28.6% (CSR+PR) | NA |
| Kawaguchi and colleagues [16] | Japan (M) | 1999-2000 | 20 | 34 | 50  (Onset) | 28.6% | 0.0% | 85.0% | 38.2% | 115 | 30% (CSR) | NA |
| Moura and colleagues [17] | Portugal (S) | 2007-2023 | 15 | 82 | 50  (Onset) | NA | 15.9% | 62.2% | 52.4% | 96 | NA | NA |
| Yaman and colleagues [18] | Turkey (S) | 2011-2022 | 13 | 101 | 50  (Age) | NA | 9.8% | 52.3% | 73.0% | 60 | NA | NA |
| Olanow and colleagues [27] | United States (S) | 1977-1979 | 12 | 12 | 55  (Age) | 25.0% | 16.6% | 75.0% | 58.3% | > 30 | 75% no medication | NA |
| MGTX Trial [4] | International (M-RCT) | 2006-2012 | 9 | 17 | 50  (Age) | NA | 0.0% | 100.0% | NA | 36 | NA | NA |
| The studies in the table are ranked based on the reported number of LOMG patients who underwent thymectomy. Abbreviation: AChR-Ab, Acetylcholine receptor antibody; B, Bicentric study; CNR, Composite neurological remission(CSR+PR+MM-0); CSR, Complete stable remission; EOMG, Early-onset myasthenia gravis; FU, Follow up; LOMG, late-onset myasthenia gravis; M, Multicentric study; MG, Myasthenia gravis; MGFA-PIS, Myasthenia Gravis Foundation of America Post-intervention Status; MM, Minimal manifestations; PR, Pharmacologic remission; RCT, Randomized controlled trial; S, Single-center study; ThX, Thymectomy.  ^a^ In the LOMG and EOMG groups who underwent surgery, the response rate was evaluated based on the MGFA-PIS. | | | | | | | | | | | | |
