# Supplementary material for: Robotic Extended Thymectomy in Late‐Onset Myasthenia Gravis: A 21‐Year Retrospective Cohort Study of 172 Patients
Source: Eur J Neurol. 2025 Nov 5;32(11):e70388. doi: 10.1111/ene.70388 (PMC12587165; doi:10.1111/ene.70388)
Supplement: Supplementary file 8 — Data S1: ene70388‐sup‐0008‐Supinfo.docx. [file ENE-32-e70388-s005.docx]

**Abbreviations in Manuscript**

AChR-Ab, Acetylcholine receptor antibody

AI, Artificial intelligence

AZA, Azathioprine

C-D, Clavien-Dindo

CI, Confidence interval

CNR, Composite neurological remission (CSR+PR+MM-0)

CSR, Complete stable remission

EOMG, Early-onset myasthenia gravis

FU, Follow up

GMG, Generalized myasthenia gravis

HR, Hazard ratio

IG, IVIg therapy

IM, Immunosuppression therapy other than prednisone (azathioprine, mycophenolate mofetil, methotrexate)

K-M, Kaplan-Meier

LOMG, Late-onset myasthenia gravis

IQR, Interquartile range

TAMG, Thymoma-associated MG

MDT, Multidisciplinary team

MG, Myasthenia gravis

MGFA, Myasthenia Gravis Foundation of America

MGFA-PIS, Myasthenia Gravis Foundation of America Post-intervention Status

MGTX, The Thymectomy Trial in Non-Thymomatous Myasthenia Gravis Patients Receiving Prednisone

MM, Minimal manifestations

MM-0, Minimal manifestations-0

NCCN, National Comprehensive Cancer Network

NT, No therapy

OAID, Other autoimmune diseases

OMG, Ocular myasthenia gravis

OR, Odds ratio

OT, Other forms of therapy (rituximab, eculizumab, efgartigimod, ravulizumab, daratumumab)

PE, Plasma exchange therapy

PR, Pharmacologic remission

RATS, Robotic-assisted thoracic surgery

RCT, Randomized controlled trial

SD, Standard deviation

ThX, Thymectomy

V-LOMG, Very late-onset myasthenia gravis

*, Significant at p＜0.05

** , Significant at p＜0.005
